# Sex Differences in Diabetes Mellitus Mortality Trends in Brazil, 1980-2012

**DOI:** 10.1371/journal.pone.0155996

**Published:** 2016-06-08

**Authors:** Thainá Alves Malhão, Alexandre dos Santos Brito, Rejane Sobrino Pinheiro, Cristiane da Silva Cabral, Thais Medina Coeli Rochel de Camargo, Claudia Medina Coeli

**Affiliations:** 1 Institute for Studies in Collective Health, Federal University of Rio de Janeiro, Rio de Janeiro, RJ, Brazil; 2 School of Public Health, University of São Paulo, São Paulo, SP, Brazil; 3 Department of Political Science, University of São Paulo, São Paulo, SP, Brazil; Garvan Institute of Medical Research, AUSTRALIA

## Abstract

**Aims:**

To investigate the hypothesis that the change from the female predominance of diabetes mellitus to a standard of equality or even male preponderance can already be observed in Brazilian mortality statistics.

**Methods:**

Data on deaths for which diabetes mellitus was listed as the underlying cause were obtained from the Brazilian Mortality Information System for the years 1980 to 2012. The mortality data were also analyzed according to the multiple causes of death approach from 2001 to 2012. The population data came from the Brazilian Institute of Geography and Statistics. The mortality rates were standardized to the world population. We used a log-linear joinpoint regression to evaluate trends in age-standardized mortality rates (ASMR).

**Results:**

From 1980 to 2012, we found a marked increment in the diabetes ASMR among Brazilian men and a less sharp increase in the rate among women, with the latter period (2003–2012) showing a slight decrease among women, though it was not statistically significant.

**Conclusions:**

The results of this study suggest that diabetes mellitus in Brazil has changed from a pattern of higher mortality among women compared to men to equality or even male predominance.

## Introduction

The prevalence of diabetes mellitus has increased considerably in the last decades and varies between communities, showing differences in environmental and genetic factors [1]. Global estimates indicate that the total number of adults aged 20–79 years with this illness will rise from 415 million in 2015 to 642 million in 2040. Likewise, prevalence will increase from 8.8% to 10.4% in the same period [1].

Some studies have found sex differences in risk factors, clinical manifestations and sequelae of diabetes mellitus and verified that prevention, detection, and treatment affect men and women differently [2–3]. Moreover, data had shown that this disease went from a pattern of higher prevalence among women compared to men to equality or even male predominance [4–6].

In Brazil, despite the increase in prevalence [7–9], this change between sexes is not clear. We observed different results between studies that assessed diabetes status through self-report questionnaires and surveys based on blood glucose measurement. Between 1986 and 1988, the only multicenter survey of national scope that was carried out, which included blood glucose screening, found that men and women had similar prevalence rates of diabetes [10]. Local studies based on blood tests also showed that differences in diabetes prevalence in the male and female populations disappear when pre-diagnosed and undiagnosed cases are evaluated [11]. However, an investigation conducted between 2010 and 2012, which included blood glucose measurement, concluded that diabetes affects more men than women [12]. On the other hand, national surveys based on the self-reported prevalence of diabetes mellitus were always higher for women than men [7–8, 13].

Mortality statistics could shed some light on the sex difference in diabetes prevalence. In Brazil, the majority of deaths occurs in hospitals [14], increasing the odds of diagnosing and reporting diabetes in the death certificate. The mortality data are thus less influenced by late diagnosis when compared to self-reported studies, still relatively frequent in this country [15]. Therefore, the aim of this study was to evaluate the pattern of mortality from diabetes mellitus by sex, from 1980 to 2012, in order to assess whether a change from female diabetes mortality predominance to a standard of equality or even male preponderance has occurred in Brazil.

## Materials and Methods

We carried out an ecological, time-series study based on official Brazilian statistics. For the calculation of diabetes mortality rates, we used the Brazilian Mortality Information System (from 1980 to 2012; available at http://www.datasus.gov.br) and population data provided by the Brazilian Institute of Geography and Statistics (IBGE, available at http://ibge.gov.br/home/). We used data from National Censuses (1980, 1991, 2000 and 2010), Population Count (1996) and estimates (for the remaining years).

We used two different approaches in order to calculate diabetes mortality rates. From 1980 to 2012, we included the death records that mention diabetes mellitus as the underlying cause. For the 2001–2012 period, we also included records in which diabetes mellitus was mentioned as an associated cause of death, either in Part I or in Part II of the cause-of-death section of the death certificate. Associated causes of death became available in the Brazilian mortality databases in 1999. However, within the initial period (1999 to 2000), the completeness of this information was low. For record selection, we used the International Classification of Diseases (ICD) codes corresponding to diabetes mellitus, as follows: 250 of the ninth revision (1980–1995); and E10 to E14 of the tenth revision (1996–2012).

We calculated both crude and age-adjusted mortality rates according to sex. We calculated the annual age-adjusted mortality rates (ASMR), with 95% confidence intervals and used the direct method [16], standardized to the world population in 2012 [17]. We considered the following groups: 20–29, 30–39, 40–49, 50–59, 60–69, 70–79 and 80 or more years old.

We applied the log-linear joinpoint regression to evaluate trends in age-standardized mortality rates and its annual percentage change (APC), with a 95% confidence interval. The joinpoint regression model considered the heteroscedasticity and autocorrelation of the random errors.

The maximum number of joinpoints was determined based on the number of data points, according to the grid search method. The final model considered the number of joinpoints statistically significant at an overall level of significance of 5% [18]. The Average Annual Percentage Change (AAPC) was also calculated to compare the estimated changes in mortality. A positive and negative AAPC indicated an increase and decrease in the mean trend, respectively [19].

ASMR and their standard errors were computed using STATA version 10.1 (Stata Corp., College Stata Station, TX; www.stata.com). Moreover, the joinpoint regression was carried out using the Joinpoint Software version 4.1.1 available from the National Cancer Institute (NCI; http://surveillance.cancer.gov/joinpoint/).

The patient records/information were anonymized and de-identified before analysis. Thus, there is no identification of individuals from our aggregate data. Moreover, the data are public and available on government websites.

The ethics committee from the Institutional Review Board of the Institute for Studies in Collective Health (IESC) at the Federal University of Rio de Janeiro (UFRJ) approved this study (CAE 48174515.0.0000.5286).

## Results

### Underlying cause of death

From 1980 to 2012, 955,455 people in Brazil aged 20 years or older died of diabetes mellitus. Women accounted for 57.7% (n = 551,016) of deaths and men, 42.3% (n = 404,439).

The ASMR from diabetes mellitus has increased since 1980, from 20.8 per 100,000 (95% CI: 20.2–21.5) to 47.6 per 100,000 (95% CI: 47.0–48.2) for men and from 28.7 per 100,000 (95% CI: 27.9–29.4) to 47.2 per 100,000 (95% CI: 46.7–47.7) for women in 2012 (Table 1).

**Table 1:** Deaths mentioned as the underlying cause and mortality rates from diabetes mellitus by sex. Brazil, 1980 to 2012.

	Male				Female			
Year of death	N	Crude rate*	ASMR*	95%CI	N	Crude rate*	ASMR*	95%CI
1980	4,150	14.1	20.8	20.2–21.5	6,120	20.1	28.7	27.9–29.4
1981	4,325	14.3	20.7	20.1–21.4	6,492	20.7	29.1	28.4–29.8
1982	4,721	15.1	21.8	21.2–22.5	6,871	21.2	29.5	28.8–30.2
1983	5,109	15.9	22.9	22.3–23.6	7,446	22.2	30.7	29.9–31.4
1984	4,952	15.0	21.3	20.7–22.0	7,408	21.5	29.4	28.7–30.0
1985	5,371	15.8	22.5	21.9–23.1	7,952	22.4	30.3	29.6–31.0
1986	5,818	16.7	23.6	22.9–24.2	8,467	23.1	31.1	30.4–31.8
1987	5,877	16.4	22.9	22.3–23.5	9,084	24.2	32.2	31.5–32.9
1988	6,512	17.8	24.9	24.2–25.5	9,630	24.9	33.0	32.3–33.7
1989	7,056	18.8	26.3	25.6–26.9	10,136	25.6	33.7	33.1–34.4
1990	7,333	19.2	26.6	26.0–27.2	10,730	26.5	34.6	34.0–35.3
1991	7,537	19.2	26.7	26.0–27.3	11,084	26.6	34.6	34.0–35.3
1992	7,888	19.6	26.7	26.1–27.3	11,739	27.4	35.0	34.4–35.6
1993	8,712	21.6	30.3	29.7–31.0	12,416	29.0	38.0	37.3–38.7
1994	9,048	22.1	31.0	30.4–31.7	13,015	30.0	39.3	38.6–40.0
1995	9,523	23.0	32.3	31.6–32.9	14,038	31.9	41.9	41.2–42.6
1996	10,574	24.1	32.5	31.9–33.1	15,319	32.8	40.8	40.2–41.5
1997	11,345	25.5	34.5	33.9–35.2	15,846	33.4	41.7	41.0–42.3
1998	11,714	26.0	35.3	34.7–36.0	16,262	33.9	42.3	41.6–42.9
1999	13,187	28.9	39.5	38.8–40.1	18,178	37.4	46.8	46.1–47.5
2000	14,775	30.1	39.4	38.8–40.1	20,279	38.6	45.0	44.4–45.6
2001	14,748	29.6	39.0	38.4–39.6	20,117	37.7	44.1	43.5–44.7
2002	15,294	30.3	40.2	39.5–40.8	21,154	39.2	45.9	45.3–46.5
2003	15,889	31.1	41.5	40.8–42.1	21,427	39.2	46.0	45.4–46.6
2004	16,853	32.6	43.5	42.8–44.2	22,214	40.1	47.2	46.6–47.8
2005	17,431	32.8	44.1	43.5–44.8	22,709	39.9	47.1	46.5–47.7
2006	19,514	36.2	49.1	48.4–49.8	25,342	43.9	52.0	51.3–52.6
2007	20,369	34.7	43.5	42.9–44.1	27,157	43.0	46.6	46.1–47.2
2008	21,880	36.8	45.5	44.9–46.1	28,380	44.5	47.2	46.7–47.8
2009	22,620	37.4	45.6	45.0–46.2	29,315	45.1	47.0	46.5–47.6
2010	23,921	38.9	46.4	45.8–47.0	30,774	46.4	46.5	46.0–47.0
2011	25,508	41.1	49.1	48.5–49.7	32,196	48.1	48.3	47.8–48.8
2012	24,885	39.8	47.6	47.0–48.2	31,719	47.0	47.2	46.7–47.7

Source: Deaths: Brazilian Mortality Information System/DATASUS; Population: National Population Censuses (1980, 1991, 2000 and 2010), the Population Count (1996), and population estimates (for the remaining years)/IBGE; International Database—World Population by Age and Sex (2012)/ U.S. Census Bureau

* Rates per 100,000; ASMR: age-standardized mortality rate for diabetes mellitus (standardized to the world population in 2012); 95%CI: 95% confidence interval

**Fig 1:**
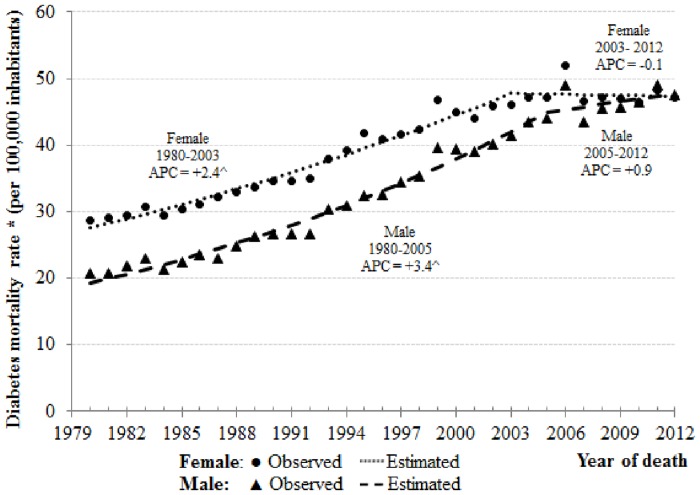
Joinpoint analysis for diabetes mellitus mortality rates (deaths mentioned as the underlying cause) by sex. Brazil, 1980 to 2012. *Age-standardized rate per 100,000 (using world population in 2012) ^ The Annual Percent Change (APC) is significantly different from zero at level of significance = 0.05.

Fig 1 shows joinpoint analysis for diabetes mellitus mortality rates by sex in Brazil, from 1980 to 2012, according to the underlying cause of death. Overall, there was an increase in the death rate trend in both sexes. However, among men and women, this trend has changed according to particular periods.

In men, between the years 1980 to 2005 and 2005 to 2012, mortality rates rose 3.4% (APC = 3.4 and 95% CI: 3.2, 3.7) and 0.9% per year (APC = 0.9, 95% CI: -0.1, 1.9), respectively. (Table 2). In women, between the years 1980 to 2003, mortality rates grew 2.4% (APC = 2.4 and 95% CI: 2.2, 2.6) per year. Nevertheless, between 2003 and 2012, there was a reduction of 0.1% per year (APC = -0.1, 95% CI: -0.7, 0.5), but it was not statistically significant (Table 2). Considering the entire analyzed period (1980–2012), the rate increased 2.9% per year in men (AAPC = 2.9, 95% CI: 2.6, 3.1) and 1.7% in women (AAPC = 1.7, 95% CI: 1.5, 1.9) (Table 2).

**Table 2:** Joinpoint analysis for diabetes mellitus mortality rates (deaths mentioned as the underlying cause) by sex. Brazil, 1980 to 2012.

	Period		ASMR			
Sex	Beginning	End	Beginning	End	APC^1^	95% CI
Male	1980	2005	20.8	44.1	3.4*	3.2–3.7
	2005	2012	44.1	47.6	0.9	-0.1–1.9
	1980	2012	20.8	47.6	2.9*	2.6–3.1
Female	1980	2003	28.7	46.0	2.4*	2.2–2.6
	2003	2012	46.0	47.2	-0.1	-0.7–0.5
	1980	2012	28.7	47.2	1.7*	1.5–1.9

*Note*: ASMR: age-standardized mortality rate for diabetes mellitus per 100,000; APC: annual percent change; 95%CI: 95% confidence interval

^1^ We computed the Average Annual Percentage Change (AAPC) over the entire period considered (1980–2012).

* The APC is significantly different from zero at level of significance = 0.05.

### Multiple causes of deaths

Considering diabetes mellitus as the underlying or associated cause of mortality from 2001 to 2012, the number of deaths was 1,076,434 (women: 603,686–56.1%; men: 472,748–43.9%). This represents a 95.2% increase when compared with the figures based only on the underlying cause of death in the same period.

The ASMR from diabetes mellitus has increased since 2001, from 76.1 per 100,000 (95% CI: 75.2–77.0) to 95.6 per 100,000 (95% CI: 94.8–96.5) for men and from 83.7 per 100,000 (95% CI: 82.9–84.6) to 93.3 per 100,000 (95% CI: 92.6–94.1) for women in 2012 (Table 3).

**Table 3:** Deaths and mortality rates from diabetes mellitus (multiple causes of death approach) by sex. Brazil, 2001 to 2012.

	Male				Female			
Year of death	N	Crude rate*	ASMR*	95%CI	N	Crude rate*	ASMR*	95%CI
2001	28,603	57.4	76.1	75.2–77.0	38.144	71,5	83.7	82.9–84.6
2002	30,472	60.4	80.5	79.6–81.5	40.412	74,8	87.8	87.0–88.7
2003	31,426	61.5	82.5	81.6–83.4	41.443	75,8	89.1	88.2–89.9
2004	34,057	65.9	88.5	87.5–89.4	44.017	79,5	93.7	92.8–94.5
2005	35,558	66.9	90.5	89.6–91.5	45.127	79,3	93.8	92.9–94.6
2006	38,080	70.6	96.1	95.2–97.1	48.636	84,3	99.9	99.0–100.8
2007	39,754	67.7	85.3	84.5–86.2	50.886	80,6	87.5	86.7–88.2
2008	42,713	71.9	89.2	88.4–90.1	54.027	84,7	90.0	89.2–90.8
2009	44,323	73.2	89.6	88.8–90.4	56.249	86,5	90.3	89.5–91.0
2010	47,538	77.3	92.3	91.5–93.2	59.772	90,1	90.4	89.7–91.1
2011	50,334	81.2	97.1	96.3–98.0	62.305	93,1	93.5	92.8–94.2
2012	49,890	79.8	95.6	94.8–96.5	62.668	92.9	93.3	92.6–94.1

Source: Deaths: Brazilian Mortality Information System/DATASUS; Population: National Population Census (2010) and population estimates (for the remaining years)/IBGE; International Database—World Population by Age and Sex (2012)/ U.S. Census Bureau

* Rates per 100,000; ASMR: age-standardized mortality rate for diabetes mellitus (standardized to the world population in 2012); 95%CI: 95% confidence interval

**Fig 2:**
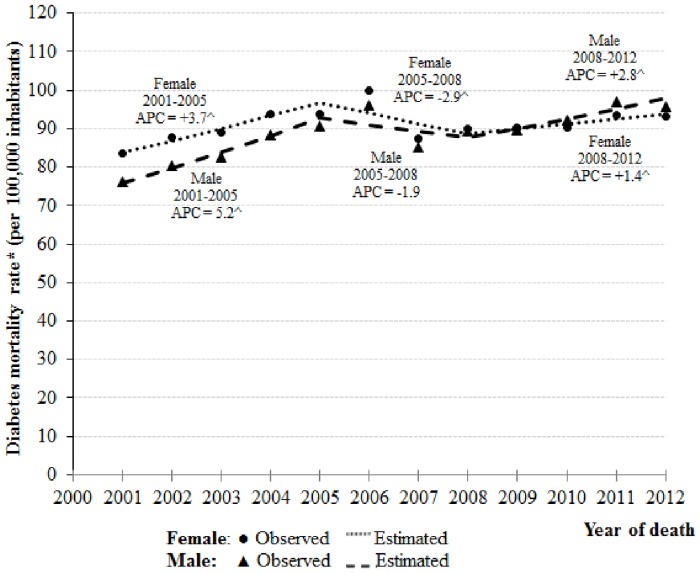
Joinpoint analysis for diabetes mellitus mortality rates (multiple causes of death approach) by sex. Brazil, 2001 to 2012. *Age-standardized rate per 100,000 (using world population in 2012) ^ The Annual Percent Change (APC) is significantly different from zero at level of significance = 0.05.

According to the joinpoint analysis (Fig 2, Table 4), in men, from the years 2001 to 2005 and 2008 to 2012, mortality rates from diabetes increased 5.2% (APC = 5.2 and 95% CI: 3.6, 6.8) and 2.8% per year (APC = 2.8, 95% CI: 1.6, 4.1), respectively. Nevertheless, between 2005 and 2008, there was a decline of 1.9% per annum (APC = -1.9 and 95% CI: -7.6, 4.2) (Table 4).

**Table 4:** Joinpoint analysis for diabetes mellitus mortality rates (multiple causes of death approach) by sex. Brazil, 2001 to 2012.

	Period		ASMR			
Sex	Beginning	End	Beginning	End	APC^1^	95% CI
Male	2001	2005	76.1	90.5	5.2*	3.6–6.8
	2005	2008	90.5	89.2	-1.9	-7.6–4.2
	2008	2012	89.2	95.6	2.8*	1.6–4.1
	2001	2012	76.1	95.6	2.4*	1.1–3.7
Female	2001	2005	83.7	93.8	3.7*	3.0–4.3
	2005	2008	93.8	90.0	-2.9*	-5.4–0.3
	2008	2012	90.0	93.3	1.4*	0.9–2.0
	2001	2012	83.7	93.3	1.0*	0.5–1.6

ASMR: age-standardized mortality rate for diabetes mellitus per 100,000; APC: annual percent change; 95%CI: 95% confidence interval

^1^ We computed the Average Annual Percentage Change (AAPC) over the entire period considered (2001–2012).

* The APC is significantly different from zero at level of significance = 0.05.

In women, mortality rates have risen 3.7% (APC = 3.7 and 95% CI: 3.0, 4.3) and 1.4% per year (APC = 1.4, 95% CI: 0.9, 2.0), from 2001 to 2005 and 2008 to 2012, respectively. Nonetheless, between 2005 and 2008, there was a decrease of 2.9% per year (APC = -2.9, 95% CI: -5.4, -0.3) (Table 4).

Between 2001 and 2012, the ASMR grew 2.4% per year (AAPC = 2.4, 95% CI: 1.1, 3.7) in men and 1.0% in women (AAPC = 1.0, 95% CI: 0.5, 1.6) (Table 4).

## Discussion

In Brazil, from 1980 to 2012, we found a marked increment in the diabetes ASMR among men and a less sharp increase in the rate among women, with the latter period (2003–2012) showing a slight decrease among women, though it was not statistically significant. This indicates a change in the sex ratio. Diabetes mellitus is frequently under-reported in mortality statistics, especially when based only on the underlying cause of death [20–21]. This study corroborates these findings. In the analysis that considered any mention of diabetes in the death certificate, we identified that in half of the cases, diabetes was reported as the underlying cause of death. Nevertheless, we observed a rising trend of ASMR from diabetes mellitus in both sexes, regardless of the approach used. This trend is a result of the interplay between the increment in diabetes incidence and prevalence, as well as in the risk of death among people with diabetes.

In Brazil, from 1986 to 1987, the self-reported prevalence of diabetes mellitus was 4.1% among the urban population aged 30–69 years (men: 3.1%; women: 4.7%) [10]. In 2013, this value was 6.2% (men: 5.4%; women: 7.0%) among individuals aged 18 years or older [13]. Considering the estimates from the World Health Organization, the national prevalence of raised blood glucose among Brazilian adults (18 + years old) increased from 7.0% (men: 7.1%; women: 6.8%) in 2010, to 7.6% (men: 8.0%; women: 7.3%) in 2014 [22].

As in other countries, the growing prevalence resulted from population aging, a rapid increase in overweight/obesity and a rise in unhealthy lifestyles [23]. The expanded access to diagnosis and treatment, resulting from a series of population health interventions implemented during the last decades, may also play a significant role. First, the creation of the Unified Health System (SUS), in 1988, based on the universal right to health care, and which, in theory, assures unrestricted access to the entire population [24]. Second, the expansion of the Family Health Strategy, which implements a model of primary care in which families living within a specific geographical area receive preventive and curative health interventions that are carried out by a multiprofessional team [25]. Finally, the implementation of health policies that seek specifically to control non-communicable diseases, such as the Brazilian Ministry of Health’s Plan for the Reorganization of Care for Arterial Hypertension and Diabetes Mellitus [26], created in 2001, and the Strategic Action Plan for Confronting Chronic Non-communicable Diseases in Brazil 2011–2022, launched in 2011 [15, 27].

This pattern of growth in mortality rates did not follow, in general, the evidence found in epidemiological studies conducted in different countries. The increasing incidence and prevalence notwithstanding, these investigations found a reduction in mortality from diabetes mellitus in recent decades [5, 28–29]. The possible explanations given this decline were: (a) decrease in mortality from coronary heart disease and stroke; (b) lower exposure to risk factors (e.g. smoking); (c) active search and screening for early detection and; (d) development of medical interventions for the reduction of blood glucose, blood cholesterol, and arterial pressure [29–30]. In Brazil, these trends are also in place, but are much more recent [23]. It is to be expected that their impact will take some time to become evident. Nonetheless, our results seem to suggest effects are already under way, especially among women, for whom the ASMR for diabetes has stabilized.

The change in the sex ratio observed in our results is consistent with recent data that shows that, in developed countries, this disease has shifted from a pattern of higher prevalence among women compared to men to equality or even male predominance [4–6]. Likewise, it supports the results found in a Brazilian investigation, which included blood glucose measurement and verified that diabetes affects more men than women [12].

Though the reasons for the change in the sex ratio remain unclear, sex-dissimilarities in biology and the different ways that men and women respond to broad contextual changes in recent years in Brazil can help us to interpret our results.

Men develop type 2 diabetes at a lower body-mass index (BMI) than women [31]. Moreover, they have higher waist circumference, greater amounts of visceral and hepatic fat and are more insulin resistant [31–33]. For that reason, the rise in diabetes mortality among Brazilian men could be associated with the higher growth in the proportion of overweight (BMI ≥ 25 Kg/m²) individuals among this group when compared with women between the periods 1974–1985 (men: 18.5%; women: 28.7%) and 2008–2009 (men: 50.1%; women: 48.0%) [34].

Additionally, women with diabetes have a greater excess risk for fatal coronary heart disease than men [35–37]. This is because women have a higher number of vascular risk factors, such as inflammatory parameters, unfavorable changes in coagulation and blood pressure, especially after menopause [35]. They also have worse results in their treatment, probably due to sex differences in the effects of drug therapy [35]. Thus, the reduction in the growth of ASMR among women could be a reflex of the decrease in cardiovascular mortality verified in Brazil in the last decade [23].

Thus far, in this article, we have examined the change in the sex ratio and its possible explanations in terms of sex. This is because the information recorded on death certificates, from which we have extracted the data for this analysis, refers to male and female sex, and not to gender. However, as the vast majority of the population is cisgender, we can consider that these groups reflect, overall, men and women. This fact enables us to consider gender disparities as possible explanations for the differences in prevalence and mortality we are discussing.

Though access to education had expanded for all Brazilians in this period, women had greater gains than men in terms of educational attainment [38]. Several studies have shown that educational attainment is inversely associated with diabetes [39–42]. Additionally, this association is also more pronounced among women than among men [39–40, 42]. A possible mechanism at play is that increases in schooling have a causal effect on maintaining a healthy BMI and waist circumference among women more so than among men [43]. This would result, for example, in the greater proportion of overweight individuals among men [44–45].

We also expect policies that expand access to care to benefit men less than women. Women utilize health care services more frequently, at least in part due to pregnancy, childbirth and a larger number of chronic conditions [11, 37]. Another possible explanation is that men value health less than women and are less likely to believe that health care is effective [11]. Therefore, it is possible that Brazilian men are accessing diagnosis at a later stage, after the onset of complications from diabetes, and when the prognosis is worse [11]. The inclusion of a gender perspective in health planning is, therefore, necessary in order to improve health care service use, especially among men, and in order to guarantee the success of the opportunistic screening strategy proposed by the American Diabetes Association [46].

Investigations using mortality statistics, such as ours, involve the analysis of data available on death certificates in large populations. As a result, when combined with other types of studies, they allow us to understand better the sex differences in diabetes mellitus in Brazil. This study could contribute to developing public programs and policies to improve diagnosis and management of diabetes based on gender differences. The cost-efficiency advantage given by this type of study makes it proper for examining scientific hypotheses that can be further tested adopting more robust research designs [22]. It is also true that their results should be considered in light of several limitations, as available below. In Brazil, the coverage and the quality of the Mortality Information System improved during the period studied, which may overestimate the increase in mortality rates [47]. The completeness of death counts rose from about 80% in 1980–1991 to over 95% in 2000. At the same time, the proportion of ill-defined causes of deaths decreased by about 53% [47]. Besides, there is potential for misclassification of diabetes type in the Brazilian Mortality Information System. As a result, approximately 90% of deaths from diabetes mellitus are unspecified, hampering the comparison between type 1 or type 2 diabetes [14]. Likewise, the growth in mortality can also be partly an effect of the modifications in the diagnostic criteria [48]. An additional limitation that should be considered is the change in the classification system for coding the cause of death. However, previous studies of comparability between ICD-9 and ICD-10 observed small differences in definitions of coding methods, which did not generate distortions in the quantity of deaths from diabetes mellitus [49].

One strength of the present study is the availability of data for a longer period. Although previous studies carried out in Brazil showed a growing trend of the mortality rate for diabetes [15], to our knowledge, this was the first study that demonstrated the changes in the sex ratio mortality for diabetes according to different periods of time. The joinpoint regression analysis allows us to determine changes in the trends of mortality rates and to recognize exactly when these changes occur and measure them [50]. Another benefit is that there is no need to pre-specify periods, avoiding bias in the investigation. For instance, in our study the years of 2005 for men and 2003 for women were identified as the joinpoints. The disadvantage is the fact that the associated time intervals could be different for each population subgroup, hampering the comparison among them [51].

## Conclusions

In conclusion, diabetes mellitus mortality rates are increasing among Brazilian men and possibly decreasing among women. The results of this study therefore indicate that this disease has changes from a pattern of higher mortality among women compared to men to equality or even male predominance. However, further investigation must be conducted to elucidate these patterns and the sex differences in diabetes outcomes.
